# Highly sensitive and rapid determination of tacrolimus in peripheral blood mononuclear cells by liquid chromatography–tandem mass spectrometry

**DOI:** 10.1002/bmc.4416

**Published:** 2018-11-14

**Authors:** Soma Bahmany, Lucia E.A. de Wit, Dennis A. Hesselink, Teun van Gelder, Nauras M. Shuker, Carla Baan, Bart C.H. van der Nagel, Birgit C.P. Koch, Brenda C.M. de Winter

**Affiliations:** ^1^ Department of Hospital Pharmacy, Erasmus MC University Medical Center Rotterdam the Netherlands; ^2^ Department of Internal Medicine, Rotterdam Transplant Group, Erasmus MC University Medical Center Rotterdam the Netherlands

**Keywords:** intracellular concentration, peripheral blood mononuclear cells, tacrolimus, UPLC–MS/MS

## Abstract

After solid organ transplantation, tacrolimus is given to prevent rejection. Therapeutic drug monitoring is used to reach target concentrations of tacrolimus in whole blood. Because the site of action of tacrolimus is the lymphocyte, and tacrolimus binds ~80% to erythrocytes, the intracellular tacrolimus concentration in lymphocytes is possibly more relevant. For this purpose, we aimed to develop, improve and validate a UPLC–MS/MS method to measure tacrolimus concentrations in isolated peripheral blood mononuclear cells (PBMCs). PBMCs were isolated using a Ficoll separation technique, followed by a washing step using red blood cell lysis. A cell suspension of 50 μL containing 1 million PBMCs was used in combination with MagSiMUS‐TDM^PREP^. To each sample we added 30 μL lysis buffer, 20 μL reconstitution buffer containing ^13^C^2^H_4_‐tacrolimus as internal standard, 40 μL MagSiMUS‐TDM^PREP^ Type I Particle Mix and 175 μL Organic Precipitation Reagent VI for methanol‐based protein precipitation. A 10 μL aliquot of the supernatant was injected into the UPLC–MS/MS system. The method was validated, resulting in high sensitivity and specificity. The method was linear (*r* ^2^ = 0.997) over the range 5.0–1250 pg/1 × 10^6^ PBMCs. The inaccuracy was <5% and the imprecision was <15%. The washing steps following Ficoll isolation could be performed at either room temperature or on ice, with no effect of the temperature on the results. A method for the analysis of tacrolimus concentrations in PBMCs was developed and successfully validated. Further research will be performed to investigate the correlation between concentrations in PBMCs and clinical outcome.

## 1 INTRODUCTION

Immunosuppressive therapy is necessary to prevent acute rejection after solid organ transplantation. The current drug regimen of first choice after organ transplantation is tacrolimus in combination with mycophenolic acid, which is often continued lifelong (Hariharan et al., 2000, Meier‐Kriesche et al., 2006). Prolonged use of tacrolimus causes considerable toxicity and the side effects negatively impact on long‐term patient and allograft survival (Hesselink & Hoorn, 2014; Lamb, Lodhi, & Meier‐Kriesche, 2011). Tacrolimus is difficult to dose owing to its narrow therapeutic window and wide inter‐individual variability in its pharmacokinetics (Kaufman et al., 2004; Matas et al., 2013). Therefore, treatment with a standard dose is not recommended and therapeutic drug monitoring (TDM) is routinely used to reach target predose concentrations in whole blood (Passey et al., 2011; Staatz & Tett, 2004).

Despite improvements in immunosuppressive treatment protocols, acute cellular rejection remains a concern, with ~10–20% of renal transplant recipients suffering from an acute rejection in the first 12 months after transplantation (Group et al., 2014; Lamb et al., 2011). This occurs even when whole‐blood tacrolimus concentrations are within the target range, suggesting that whole‐blood concentrations do not accurately reflect the pharmacological effect (Bouamar et al., 2013; Capron, Haufroid, & Wallemacq, 2016). The receptor of tacrolimus is the 12 kDa FK506 binding protein (FKBP12). The tacrolimus‐FKBP12 complex in turn binds to calcineurin and blocks the activation of this calcium/calmodulin‐activated phosphatase within the T‐lymphocyte (Griffith et al., 1995. However, erythrocytes also have a high concentration of FKBP12 and tacrolimus is extensively distributed within the red blood cell compartment (Biagiotti et al., 2011). Approximately 80% (range 70–95%) of tacrolimus measured in whole blood is distributed in erythrocytes, where it has no immunosuppressive effect.

Since the site of action of tacrolimus is within the lymphocyte, it seems reasonable to assume that the tacrolimus concentration within the lymphocyte is more relevant than the whole‐blood concentration when predicting treatment efficacy. Recent studies support this assumption (Capron et al., 2016; Han et al., 2016). Several methods have been developed and validated for quantification of tacrolimus in peripheral blood mononuclear cells (PBMCs;i.e., lymphocytes and monocytes; Capron et al., 2007; Capron et al., 2009; Lemaitre, Antignac, & Fernandez, 2013; Pensi et al., 2015). However, these analytical methods are complicated and time consuming compared with whole blood tacrolimus assays. The PBMC isolation in these methods is in general performed using the Ficoll separation technique on ice, to prevent tacrolimus efflux from the cells. In some centers, purification of the cell pellet is performed to decrease contamination with erythrocytes. The need for sample purification is, however, not clear. Thereafter, 1–10 million PBMCs are used for the complicated and time‐consuming sample preparation and the tacrolimus concentrations are measured on immunoassay or UPLC–MS/MS (Capron et al., 2007; Capron et al., 2009; Lemaitre et al., 2013; Pensi et al., 2015). Here, a more rapid and sensitive UPLC–MS/MS assay for the measurement of the tacrolimus concentration in PBMCs is described. In this method we improved the cell isolation, investigated the need for isolation on ice and the effect of erythrocyte purification of the cell pellet, and developed a new, more rapid and sensitive sample preparation for UPLC–MS/MS analysis.

## 2 MATERIALS AND METHODS

### 2.1 Chemicals and reagents

Tacrolimus and ammonium acetate were purchased from Sigma‐Aldrich Chemie B.V. (Zwijndrecht, the Netherlands) and ^13^C^2^H_4_‐tacrolimus from Alsa Chim (Illkirch‐Graffenstaden, France). MagSiMUS‐TDM^prep^ Kit from MagnaMedics containing MagSiMUS‐TDM^prep^ Type I Particle Mix (Beads), Reconstitution Buffer IS, Organic Precipitation Reagent VI (OPR VI) for methanol‐based protein precipitation and lysis buffer for whole blood were purchased from MagnaMedics Diagnostics B.V. (Geleen, the Netherlands). Water was purified using a MilliPore Advantage A10 System. Methanol and formic acid were purchased from Biosolve BV (Valkenswaard, the Netherlands). Human tacrolimus‐free PBMCs were isolated using Ficoll‐Paque Plus (GE Healthcare Bio‐Sciences AB, Uppsala, Sweden) from buffy coats in citrate blood obtained from Blood bank Sanquin (Rotterdam, the Netherlands). Red blood cell lysis buffer from eBioscience (Affimetrix Inc., San Diego, CA, USA) was used for purification. Cells were counted using a SysmexXOP‐300 cell counter (Sysmex Corporation, Kobe, Japan).

### 2.2 Stock solutions, calibration standards, quality control samples and internal standard

The calibration standards and quality controls were prepared using different stock solutions and tacrolimus‐free PBMCs. Stock solutions of tacrolimus and ^13^C^2^H_4_‐tacrolimus (500 mg L^−1^) were prepared by dissolving 25 mg in 50 mL of methanol. Stock solutions were stored at −80°C. Two working solutions were prepared by diluting the stock solutions with human tacrolimus‐free PBMCs isolated from buffy coats. The concentrations of the lower limit of quantitation (LLOQ) standard and calibration standards are given in Table 1 and the concentrations of the quality controls are given in Table 2. Calibration standard 8 and quality control high (QC H) were prepared by diluting the working solution with human tacrolimus‐free PBMCs. Calibration standards 5–7 were prepared by diluting standard 8; calibration standards 3 and 4 were prepared by diluting standard 5; calibration standard 2 was prepared by diluting standard 3; and calibration standard 1 was prepared by diluting standard 2. All calibration standards were diluted with human tacrolimus‐free PBMCs. Quality controls low and medium (QC L and QC M) were prepared by diluting QC H with tacrolimus‐free human PBMCs. A 50 μL aliquot of the prepared standards and quality control samples were transferred into 1.5 mL safe‐lock Eppendorf tubes and stored at −80°C prior to analysis.

**Table 1:** Concentrations of the calibration standards

Analyte	LLOQ (μg L^−1^)	S1 (μg L^−1^)	S2 (μg L^−1^)	S3 (μg L^−1^)	S4 (μg L^−1^)	S5 (μg L^−1^)	S6 (μg L^−1^)	S7 (μg L^−1^)	S8 (μg L^−1^)
Tacrolimus	0.1	0.1	0.5	1.0	2.5	5.0	10	15	25

**Table 2:** Concentrations of the quality controls

Analyte	QC L (μg L^−1^)	QC M (μg L^−1^)	QC H (μg L^−1^)
Tacrolimus	0.4	8.0	20

QC L, Quality control low; QC M, quality control medium; QC H, quality control high.

### 2.3 PBMC isolation from whole blood

The PBMC isolation for patient samples was performed using 3–4 mL heparinized blood. These samples were centrifuged for 7 min at 350***g***. The upper part of the plasma layer containing platelets was removed, followed by resuspension of the residue with the same volume of phosphate‐buffered saline (PBS). The resuspended blood was pipetted in 12 mL Leucosep tubes filled with 3 mL Ficoll‐Paque Plus. Samples were centrifuged for 20 min at 850***g*** without brakes. The PBMC layer was isolated carefully and washed with PBS at room temperature. After isolation of the PBMC pellet, a lysing step was performed by adding red blood cell (RBC) lysis buffer. After this lysing step the cells were washed twice in PBS. The cells were pelleted by centrifugation 7 min at 750***g***. After the centrifugation step, the cells were resuspended in PBS. The whole isolation procedure was performed at room temperature. After isolation, the PBMCs were counted and snap frozen in liquid nitrogen and stored at −80°C in aliquots of 1 × 10^6^ cells per vial.

#### 2.3.1 Isolation at room temperature

To investigate the need to isolate PBMCs on ice to prevent the tacrolimus efflux, we compared the results of both methods. Six different samples from renal transplant patients were split into two parts after isolation of the PBMC with Ficoll‐Paque Plus at room temperature. One part was further isolated on ice and the other part at room temperature. Both samples were analyzed using the described method and the results were compared.

#### 2.3.2 Washing steps for red blood cell lysis

To purify the isolated PBMCs from disturbing red blood cells, a washing step with RBC lysis buffer was evaluated according to the manufacturer's instructions. At first, the influence of adding one washing step was tested. Furthermore, to investigate the number of washing steps needed to purify the sample a comparison between one washing steps and up to three washing steps was performed. Ten whole blood samples from renal transplant patients, containing tacrolimus, were prepared for analysis after one, two and three washing steps. The results were compared to find the minimum number of washing steps needed to eliminate the disturbing red blood cells from the isolated PBMC's. Cells were counted using a SysmexXOP‐300 cell counter.

### 2.4 Sample preparation

For the sample preparation, the reagents kit containing MagnaMedics Type I Particle Mix Beads, reconstitution buffer IS, organic precipitation reagent VI and lysis buffer were used. This sample preparation is based on paramagnetic beads which eliminates interfering proteins, phospholipids and salts from whole blood, plasma and serum prior to analysis. The protein precipitation is collected through magnetic separation. This results in fast sample preparation. This sample preparation was equal for the calibration standards, the quality control samples and the patient samples. Aliquots of 50 μL, containing 1 × 10^6^ cells, were incubated for 1 min with 30 μL lysis buffer to complete lysis of the cells spiked with 20 μL of the internal standard solution (10 μg L^−1^ tacrolimus ^13^C^2^H_4_), 40 μL of the MagnaMedics Beads and 175 μL OPR VI, and mixed by vortexing for 10 s. Samples were then centrifuged at 1811***g*** for 5 min. A 200 μL aliquot of the supernatant was transferred to an autosampler insert vial. A volume of 10 μL was injected into the LC system. The concentration measured in the sample was reported in μg L^−1^. This concentration was multiplied 50 times to calculate the amount of tacrolimus per picogram in a pellet of 50 μL containing 1 million PBMCs.

### 2.5 Instrumentation

A Waters Acquity UPLC–MS/MS system (Waters Corp., Milford, MA, USA) consisting of an Acquity binary solvent manager (chromatographic pump), a sample manager (autosampler) and a column manager was used. The UPLC was connected to a Waters TQ‐S micro mass spectrometer with a triple quadrupole. The software programs Masslynx™ V4.1 and Targetlynx V4.1 were used for data processing.

#### 2.5.1 UPLC conditions

Chromatographic separation was performed on a reversed phase Waters Acquity UPLC BEH C_18_ column (1.7 μm, 50 × 2.1 mm) at a temperature of 60°C. A gradient elution was applied using mobile phase consisting of solvent A, 2 mm ammonium acetate and 0.1% formic acid in 1 L milliQ water, and 2 mm ammonium acetate and 0.1% formic acid in 1 L methanol as solvent B. The flow rate was set at 0.5 mL min^−1^. The initial condition was 45% solvent A and 55% solvent B. Solvent B increased to 70% in 0.6 min, then rapidly changed to 90% B in 0.1 min, then solvent B increased to 100% in 0.1 min and finally the ratio of solvent A:B was 45:55 to equilibrate at starting conditions for 2.2 min. The total runtime was 3 min. The injection volume was 10 μL.

#### 2.5.2 MS/MS conditions

To determine the optimal MS settings for tacrolimus and the internal standard tacrolimus ^13^C^2^H_4,_ solutions of each compound were prepared with a concentration of 1 mg L^−1^ in methanol. These solutions were directly injected into the MS without chromatographic separation. Parameters as the cone voltages and collision energies were optimized for each compound. The product ion with the highest sensitivity was selected as the quantifier.

The MS was operated in the positive ion mode with a capillary voltage of 1.0 kV, source block temperature of 150°C and desolvation temperature 350°C, and desolvation gas flow was delivered at 900 L/h. The collision was performed by using argon gas and the collision cell pressure was 3.4 × 10^−3^ mbar. The dwell time for each transition was 50 ms. Data acquisition was performed via multiple reaction monitoring. The optimal settings were obtained by infusion experiments with a mixture of 1 μg mL^−1^ of tacrolimus and ^13^C^2^H_4_‐tacrolimus in methanol. The optimized settings for the multiple reaction monitoring of each analyte are summarized in Table 3.

**Table 3:** MS/MS settings

Analyte	Parent ion (*m*/*z*)	Product ion (*m*/*z*)	ESI mode	Cone voltage (V)	Collision energy (eV)
Tacrolimus	821.6	768.5	+	31	18
Tacrolimus ^13^C–^2^H_4_	826.6	773.5	+	31	18

### 2.6 Validation of the LC–MS/MS method

The validation of the method was based on the Food and Drug Administration guidelines for bioanalytical validations, revised 2001 (US Department of Health and Human Services, 2001). The following validation parameters were investigated.

#### 2.6.1 Linearity

To investigate the linearity of the method, a calibration curve of each analyte was prepared and analyzed containing eight calibration standards in duplicate. The calibration curve defines the relation between the concentration of the analyte and the ratio of the response of the analyte and the response of the internal standard. The calibration curve consists of a blank sample without internal standard and a zero sample (blank with internal standard), and eight nonzero samples covering the expected range, including LLOQ. Each standard was prepared and analyzed in duplicate. The correlation coefficient (*r*) has to be at least 0.995 for tacrolimus (US Department of Health and Human Services, 2001).

#### 2.6.2 LLOQ and ULOQ

The LLOQ was determined by measuring six replicates of the LLOQ standard which has the same concentration as the lowest standard on the calibration curve. The precision of the calculated concentration should be <20% and the accuracy should be between 80 and 120%. The highest standard on the calibration curve was used to decide on the upper limit of quantitation (ULOQ). The precision of the ULOQ should be <15% and the accuracy between 85 and 115% of the theoretical concentration (US Department of Health and Human Services, 2001).

#### 2.6.3 Accuracy

The accuracy was determined by measuring three concentrations, QC H, QC M and QC L, 6‐fold on the same day. The bias should not exceed 15% and the relative standard deviation should be within 15% (US Department of Health and Human Services, 2001).

#### 2.6.4 Intra‐ and inter‐day precision

The intra‐ and inter‐day precision was calculated by measuring six replicates at three concentration levels (QC L, M and H) in duplicate. The intra‐day precision was measured on the same day and the inter‐day precision was measured on six different days. The accuracy should be between 85 and 115% and the precision within the 15% (US Department of Health and Human Services, 2001).

#### 2.6.5 Stability

To investigate the autosampler stability, three QC levels were stored at 15°C after the first injection, for 24, 48, 72, 96 and 120 h. The concentrations were compared with the concentrations of the QCs at the first time. The recovery should be between 90 and 110%. The long‐term stability of stored calibration standards and QC samples in the freezer (−80°C) was investigated after a period of 12 months. The recovery should be between 90 and 110%.

#### 2.6.6 Matrix effects

To investigate the matrix effects, the method of Matuszewski (Matuszewski, Constanzer, & Chavez‐Eng, 2003) was used. Five different batches of human tacrolimus‐free PBMCs were collected. Three sets of samples were prepared (sets A–C). In set A, six different samples were prepared in milliQ water; two blanks and QC L and QC H. In set B, blanks, QC L and QC H were prepared in five different human tacrolimus‐free PBMCs. Tacrolimus was added after sample preparation. In set C, blanks, QC L and QC H were prepared in five different human tacrolimus‐free PBMCs, and tacrolimus was added before sample preparation. Blanks, QC L and QC H for each set were prepared in duplicate. Recovery was defined as the ratio of the results of set B and C (C/B × 100%). Process efficiency is the ratio from set A and set C (C/A × 100%). Matrix effects were defined by the ratio of set A and set B (B/A × 100%). Matrix effects, recoveries and process efficiencies should be between 80 and 120%.

## 3 RESULTS

### 3.1 Sample preparation

#### 3.1.1 Isolation at room temperature

The differences between isolation of PBMCs at room temperature and on ice are presented in Table 4. No structural differences as a result of cell efflux were seen. The median difference was 0.15% (*p* = 0.625).

**Table 4:** Differences between isolation of peripheral blood mononuclear cells (PBMCs) at room temperature and on ice

Sample	Tacrolimus (pg/million cells)		Difference (%)
	Room temperature	On ice	
1	92	86	7.0
2	58	68	−14.6
3	43	47	−7.1
4	35	30	14.3
5	55	36	50.0
6	42	45	−6.7

#### 3.1.2 Washing steps for red blood cell lysis

Without an RBC lysis washing step some samples showed a light red color, which is probably due to the presence of red blood cells in the pellet. A first washing step using RBC lysis buffer resulted in a decrease in the concentration of intracellular tacrolimus of 38–58%. As a consequence, the differences between one, two and three washing steps were compared (Table 5). The average ratio between one and three washing steps was 0.9 (SD 0.12). We concluded that one washing step using RBC lysis buffer was enough to purify the PBMC pellet.

**Table 5:** Difference between one and three red blood cell (RBC) washing steps

Sample	Tacrolimus (pg/million cells)		Ratio 1/3
	1× lysis	3× lysis	
1	160	189	0.85
2	130	133	0.98
3	112	112	1.00
4	75	96	0.78
5	57	56	1.02
6	34	39	0.87
7	46	52	0.88
8	56	81	0.69
9	37	43	0.86
10	42	40	1.05

### 3.2 Validation

The RSD of accuracy, intra‐ and inter‐day precision data were within the requirement of an RSD <15%. The results are shown in Table 6.

**Table 6:** Validation results

Analyte	QC	Concentration (μg L^−1^)	Accuracy RSD (%)	Intra‐day precision RSD (%)	Inter‐day precision RSD (%)
Tacrolimus	L	0.4	−4.5	10.4	11.7
	M	8.0	−1.2	6.4	6.5
	H	20	1.5	3.6	5.8

#### 3.2.1 Linearity

The calibration curve of tacrolimus was successfully validated; the correlation coefficient (*r*) was 0.999 over the concentration range of 0.1–25 μg L^−1^ fitted by a 1/*x* weighting factor and excluding the origin. This concentration range corresponds to 5–1250 pg/L × 10^6^ cells. We saw that the concentrations of the clinical samples were within this calibration range.

#### 3.2.2 LLOQ and ULOQ

**Figure 1:**
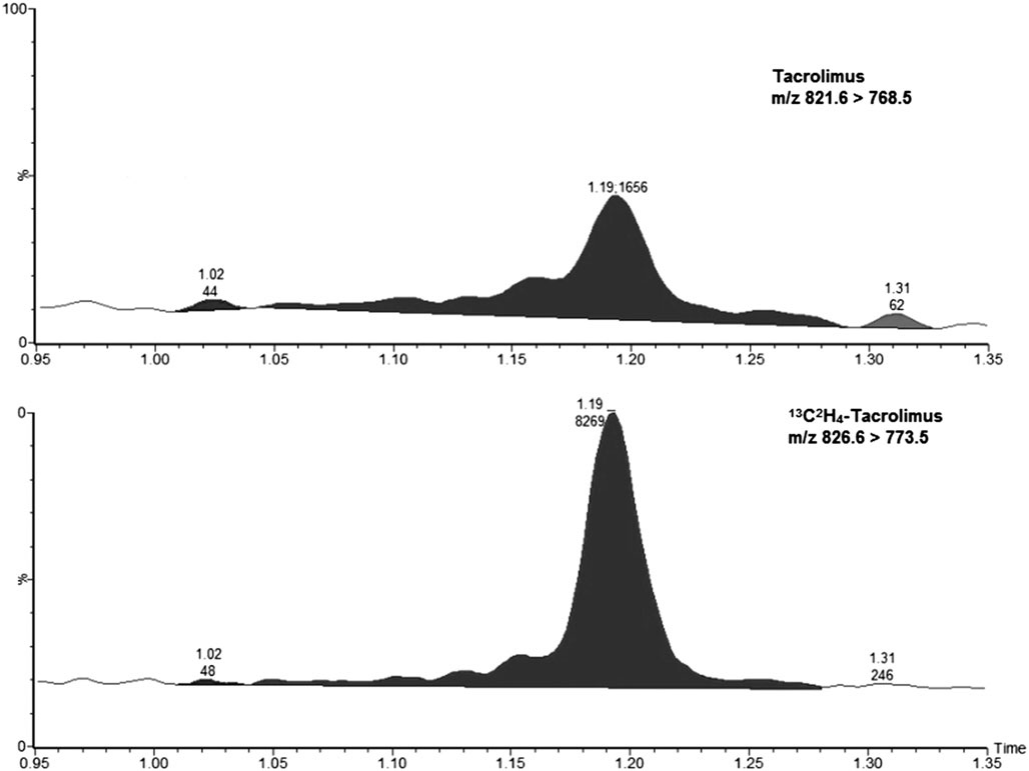
Chromatogram of the LLOQ standard (0.10 μg L^−1^) of tacrolimus with the internal standard ^13^C^2^H_4_‐tacrolimus_._ The retention time and peak‐area are given on top of the peak

The precision of the LLOQ standard 0.1 μg L^−1^ was −5% with an RSD of 11% which is within the requirement of 20% for the precision and 15% for the RSD. The ULOQ was determined as 25 μg L^−1^. The LLOQ concentration corresponds to 5 pg/L × 10^6^ cells and the ULOQ concentration corresponds to 1250 pg/L × 10^6^ cells. The corresponding chromatogram is presented in Figure 1.

#### 3.2.3 Stability

The results after 24 h in the autosampler at 15°C were not within the requirement. The recoveries of QC L were not within the requirements (89.7 and 87.5%). The recoveries of QC M (101.6 and 101.3%) and QC H (99.3 and 101.5%) were within the requirements. Extracts must be measured within 24 h.

The recovery of three QC levels after 12 months of storage were within the requirements. Calibration standards and QC samples were stable for at least 12 months at −80°C.

#### 3.2.4 Matrix effects

Matrix effects, recoveries and process efficiency were between 80 and 120% for QC H. QC L did not meet the requirements. Coefficients of variation were <5% for both QC L and QC H. The results are presented in Table 7.

**Table 7:** Matrix effects

	Set	QC L	QC H
Matrix effect (%)	B/A	136	112
Recovery (%)	C/B	45	101
Process efficiency (%)	C/A	61	112
CV (%) *n* = 10	B	0.8	1.5
CV (%) *n* = 10	C	2.3	4.0

## 4 CLINICAL APPLICATION

**Figure 2:**
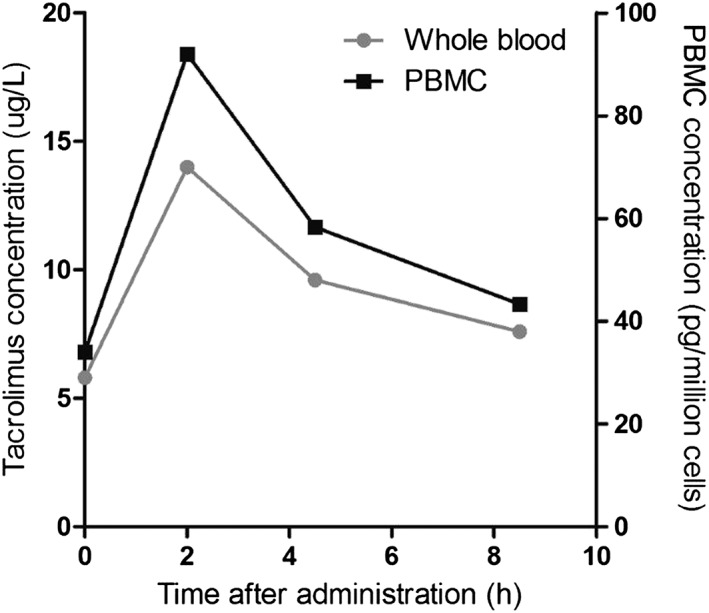
Tacrolimus concentration–time profile in whole blood vs. peripheral blood mononuclear cells (PBMCs)

To investigate whether the described assay can be used to determine the intracellular concentration of tacrolimus in isolated PBMCs for clinical purposes, a pilot experiment was performed in a renal transplant recipient. For this experiment, 4 blood samples taken just before, and 2, 4 and 8 h after oral tacrolimus administration were used. In Figure 2 the tacrolimus whole blood concentrations compared with the intracellular concentrations over a dose interval are presented. To measure the whole blood concentrations, a method published by Waters was used (Annesley et al., 2013). The whole blood/PBMC concentration ratio remained stable between 0.15 and 0.17 over this concentration–time profile.

For the experiments testing the separation of PBMCs at room temperature and the number of washing steps with RBC lysis needed, we used samples from renal transplant patients treated with tacrolimus. The assay resulted in reproducible results in these patient samples.

## 5 DISCUSSION

The development, optimization and validation of an accurate and specific method for the determination of the concentration of tacrolimus in PBMCs is described. The method described is faster and has a lower or equal LLOQ compared with other methods that were previously described in literature (Capron et al., 2007; Capron et al., 2009; Lemaitre et al., 2013; Pensi et al., 2015). This method can be used to quantify tacrolimus concentrations in PBMCs for clinical purposes and it may outperform traditional TDM using whole blood concentrations (Annesley et al., 2013) when predicting the clinical response to tacrolimus therapy of transplant recipients.

### 5.1 PBMC isolation

During the development of our assay, we investigated several aspects. First, we investigated the need to isolate PBMCs on ice, which is supposed to prevent efflux of tacrolimus during the isolation procedure. In our comparison with isolation at room temperature we did not see this supposed decrease in concentration. As a consequence, the isolation of PBMCs can be performed at room temperature, which facilitates this complicated process compared with other methods (Capron et al., 2007; Capron et al., 2009; Lemaitre et al., 2013; Pensi et al., 2015). The results are shown in Table 4.

After isolation of PBMCs using Ficoll technique, a slight red color was seen in some pellets. As tacrolimus distributes ~80% to red blood cells, this might disturb the analysis. Addition of a washing step with RBC lysis resulted in a decrease in tacrolimus concentration of 38–58% in these samples, indicating that an additional purification step to lose these red blood cells is needed. One washing step was the optimum, as we did not see any structural differences after addition of additional washing steps, which is shown in Table 5. Pensi et al. (2015) used ammonium salt solution to purify the sample. Other methods did not perform a purification washing step (Capron et al., 2007; Capron et al., 2009; Lemaitre et al., 2013).

### 5.2 Sample preparation and measurement

Several methods have been published (Capron et al., 2007; Capron et al., 2009; Lemaitre et al., 2013; Pensi et al., 2015) to determine the concentration of tacrolimus in PBMCs but none of these methods makes use of an internal standard which is identical in physical and chemical properties to the analyte. This method is unique with tacrolimus ^13^C^2^H_4_ as internal standard for the determination of tacrolimus in PBMCs. In comparison with our method, the published methods contain a time‐consuming sample preparation, because solid‐phase extraction and evaporation combined with a reconstitution step was applied. Besides the time‐consuming sample preparations, higher sample volumes were used of at least 500 μL up to a maximum of 1.1 mL. The amount of cells at each session in earlier published methods varied from 1 × 10^6^ and 6 × 10^6^ to 1 × 10^7^ cells. In that respect, our method is at the lower end of the range using 1 × 10^6^ cells (Capron et al., 2007; Capron et al., 2009; Lemaitre et al., 2013; Pensi et al., 2015).

The injection volumes also varied from 20 to 25 μL, which is more than in our method. The present method is also more sensitive, as the earlier published methods have a higher or equal LLOQ, even though they use a higher sample volume, higher concentration of the PBMCs and higher injection volume. The sample preparation of the other published methods also includes also a solid‐phase extraction and evaporation step, which could decrease the recovery of the analyte. To our knowledge, this method is the first validated LC–MS/MS method to determine tacrolimus in PBMCs without a time‐consuming sample preparation step, including a sample preparation with MagnaBeads, a lower sample volume, lower injection volume and a very short total runtime.

Despite the good results of this method, a recommendation can be made concerning the autosampler stability. The recovery of QC L did not meet the requirements after 24 h. Adjustment of the autosampler temperature to 10 or 5°C could be a solution to meet the recovery requirements. The stability during the sample preparation has not been determined, because in our setting samples are always prepared and analyzed within 1 h after sample collection.

Matrix effects experiments showed higher responses for QC L in set B (when tacrolimus was added after sample preparation). This may be caused by concentration loss of tacrolimus during the sample preparation. This gives a larger deviation at the lower concentrations in comparison with higher concentrations (QC H). In practice, this will not cause a problem, because tacrolimus is added before sample preparation for calibration standards and QCs, which means that there will be a correction for any loss of tacrolimus concentration during the sample preparation. Coefficients of variation were <5% for both QC L and QC H.

### 5.3 Clinical application

The data obtained from the renal transplant patient suggests that the dynamics of the tacrolimus concentrations in PBMCs run parallel to the pharmacokinetic profile in whole blood. We did not observe a delay in reaching the maximum concentrations in PBMCs in this patient. Apparently, tacrolimus reaches the interior of PBMCs quite easily, either by diffusion or by active transport. Possibly by drawing samples at closer time intervals, for example every 15 min, a more subtle delay in reaching concentrations in PBMCs might be detected. The ratio of the intracellular and whole blood tacrolimus concentration differs between patients (see Figure 2). These results would suggest that the ratio determined for one timepoint can be extrapolated to the whole AUC. However, more research is needed to confirm this finding and investigate if this ratio could be extrapolated within one patient over a longer time period.

## 6 CONCLUSION

In conclusion, the validation and development of this rapid and sensitive LC–MS/MS method for the measurement of tacrolimus concentrations in PBMCs was successfully finalized. The isolation of PBMCs can be performed at room temperature without consequences for the tacrolimus concentration. A red blood cell washing step has to be performed to purify the sample from contamination of tacrolimus bound to erythrocytes. In comparison with other assays, the method presented here is the first one without a time‐consuming sample preparation after the isolation of the PBMCs, using MagnaBeads sample preparation with also a high sensitivity (LLOQ = 0.1 μg L^−1^ which corresponds to 5 pg/L × 10^6^ cells). Finally, our method has a short runtime (3 min), which is very important for clinical implementation.
